# Supplementary material for: Morphological and Genetic Characterization of Eggerthella lenta Bacteriophage PMBT5
Source: Viruses. 2022 Jul 22;14(8):1598. doi: 10.3390/v14081598 (PMC9394477; doi:10.3390/v14081598)
Supplement: Supplementary file 1 [file viruses-14-01598-s001.zip › Sprotte et al. Suppl. Figure S2 01.07 final.pptx]

## Slide 1
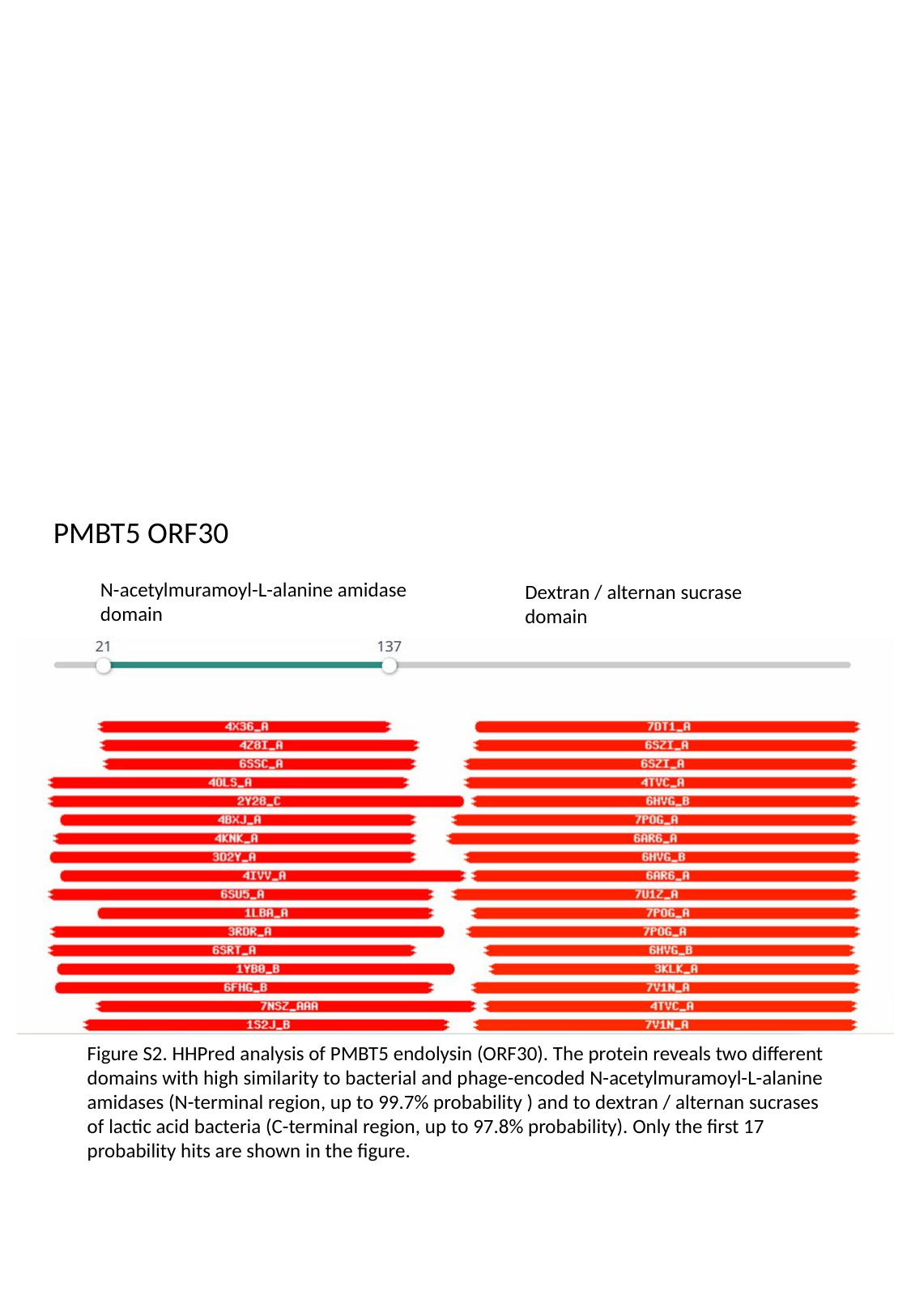

PMBT5 ORF30
N-acetylmuramoyl-L-alanine amidase
domain
Dextran / alternan sucrase
domain
Figure S2. HHPred analysis of PMBT5 endolysin (ORF30). The protein reveals two different domains with high similarity to bacterial and phage-encoded N-acetylmuramoyl-L-alanine amidases (N-terminal region, up to 99.7% probability ) and to dextran / alternan sucrases of lactic acid bacteria (C-terminal region, up to 97.8% probability). Only the first 17 probability hits are shown in the figure.
