# Supplementary material for: Morphological and Genetic Characterization of Eggerthella lenta Bacteriophage PMBT5
Source: Viruses. 2022 Jul 22;14(8):1598. doi: 10.3390/v14081598 (PMC9394477; doi:10.3390/v14081598)

Supplementary Figure S1. Workflow for the isolation of phages for anaerobic bacteria. For further details see materials and methods.

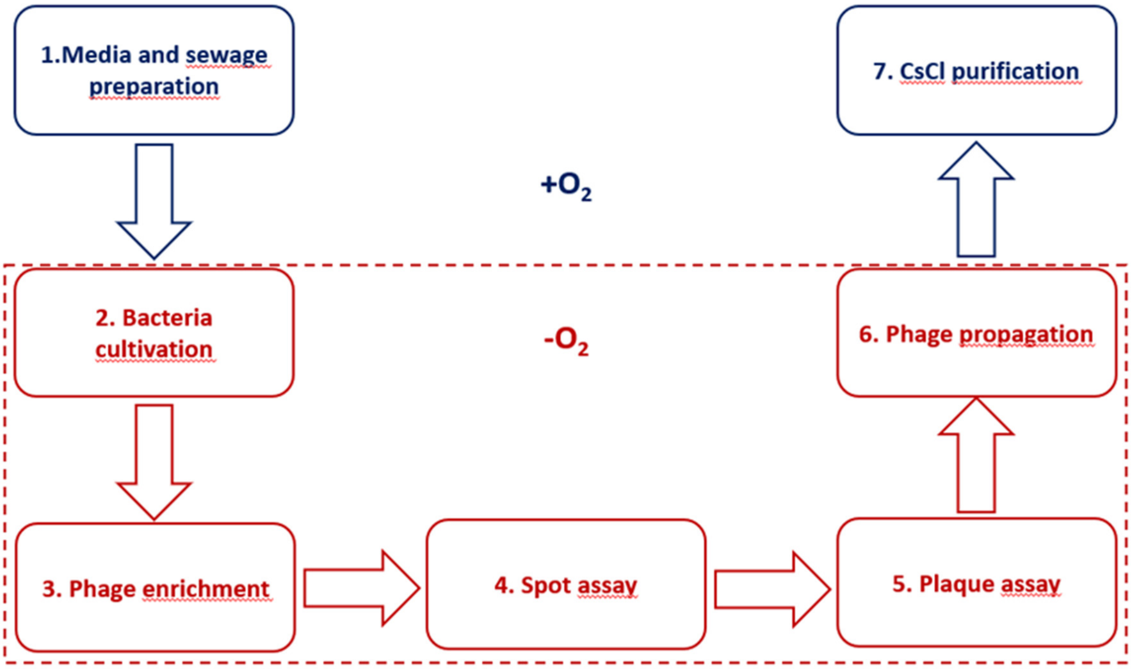

Supplement: Supplementary file 1 [file viruses-14-01598-s001.zip › Sprotte Supplementary Figure S1 01.07. final.pdf]
