# Supplementary material for: Morphological and Genetic Characterization of Eggerthella lenta Bacteriophage PMBT5
Source: Viruses. 2022 Jul 22;14(8):1598. doi: 10.3390/v14081598 (PMC9394477; doi:10.3390/v14081598)
Supplement: Supplementary file 1 [file viruses-14-01598-s001.zip › Suppl Table S1 ORFPredicted functionStrandPosition_01.07 final.pdf]

Supplementary Table S1. Overview of the 44 putative ORFs in the genome of phage PMBT5 with their predicted function and the best match result in the databases using BlastP and HHpred with significant probability hits. Proteins indicated in bold were identified as structural proteins by UHPLC-MS-MS in the manuscript.

ORF	Predicted function	Strand	Position (nt)	Length	conserved domain (acc.)	Best match BlastP [taxon]	E-Value / identity (%)	First probability hit (HHpred)	E-Value / Probability
1	Hypothetical protein	+	32..403	372	-	hypothetical protein DXC97_02530 [*Lachnospiraceae* bacterium TF09-5]	7e-12 / 37.38		
2	Large subunit terminase	+	421..1641	1221	terminase large subunit (pfam04466)	phage terminase large subunit [*Oscillospiraceae* bacterium]	1e-156 / 54.57	Large subunit terminase Deep sea thermophilic phage D6E	5.3e-43 / 100
3	Hypothetical protein	+	1638..2006	369	-	hypothetical protein [*Sphingobium* sp.]	2e-110 / 33.65		
4	**Portal protein**	+	2003..3385	1383	-	TPA: MAG TPA: portal protein [*Siphoviridae* sp.]	1e-163 / 51.18	Portal protein, *Bacillus subtilis* phage SPP1	1.9e-25 / 99.95
5	Type I R/M methyltransferase subunit	+	3382..4230	849	-	hypothetical protein [*Bifidobacterium pseudocatenulatum*]	2e-04 / 60.47	Type I R/M methyltransferase subunit, *Caldanaerobacter subterraneus*	8e-5 / 97,96
6	Hypothetical protein	+	4223..4357	135	-	hypothetical protein F368_gp09	4e-30 / 98.18		
7	ARNA	+	4354..4506	153	-	-		ARNA *Sulfolobus acidocaldarius*	1e-2 / 95,95
8	Minor capsid protein	+	4475..5620	1146	Mu protein F like protein (pfam04233)	TPA: MAG TPA: minor capsid protein [*Siphoviridae* sp.]	5e-96 / 74,46		
9	Repeat five residue (Rfr) protein	+	5932..6417	486	YjbI (COG1357) [Salmonella enterica]	pentapeptide repeat-containing protein	6e-36 / 62.42	Repeat five residue (Rfr) protein, *Cyanothece*	1.8e-18 / 99.76
10	Hypothetical protein	+	6431..6646	216	-	hypothetical protein [*Eggerthella lenta*]	3e-06 / 39.44		
11	Restriction alleviation protein	+	6688..7446	759	Restriction alleviation protein Lar (pfam14354)	Lar family restriction alleviation protein [*Eggerthella lenta*]	8e-28 / 80.95		
12	SHOCT domain-	+	7461..8138	678	-	SHOCT domain-containing protein	6e-44 / 53.15		

	containing protein					[*Gordonibacter massiliensis*]			
13	Hypothetical protein	+	8147..8332	186	-	hypothetical protein [*Lancefieldella rimae*]	8e-11 / 45.00		
14	Hypothetical protein	+	8427..8756	330	-	hypothetical protein [Clostridia bacterium]	7e-40 / 61.68		
15	Minor structural protein	+	9441..10013	573	MARTX_Nterm (NF012221)	TPA: MAG TPA: hypothetical protein [*Siphoviridae* sp.]	1e-14 / 32.98		
16	**Major capsid protein MCP**	+	10024..10899	876	-	hypothetical protein [unclassified *Ruminococcus*]	4e-154 / 73.63	Major capsid protein *Prochlorococcus* phage P-SSP7	2.7e-15 / 99,69
17	Head-to tail-interface	+	11000..11332	333	gp6_gp15_like (cl12049)	TPA: MAG TPA: putative head-tail [*Siphoviridae* sp.]	1e-15 / 37.50	Head-to tail-interface, *Bacillus subtilis* phage SPP1	1.2e-3 / 97,69
18	Hypothetical protein	+	11323.. 11787	465	-	TPA: MAG TPA: hypothetical protein [*Siphoviridae* sp.]	4e-34 / 41.56	phage-like element PBSX protein xkdH	4.6e-2 / 96
19	Minor capsid protein	+	11790..12128	339	Minor_capsid_2 (pfam11114)	TPA: MAG TPA: Minor capsid protein [*Siphoviridae* sp.]	2e-30 / 50.94		
20	Tail-to-head joining protein	+	12128..12559	432	-	TPA: MAG TPA: Minor capsid protein from bacteriophage [*Siphoviridae* sp.]	3e-54 / 57.45	Tail-to-head joining protein, *Bacillus subtilis* phage SPP1	1.5 / 93,84
21	**Major tail protein MTP**	+	12569..13075	507	-	TPA: MAG TPA: hypothetical protein [*Siphoviridae* sp.]	3e-42 / 53.10	Major tail protein MTP, *Escherichia coli* phage lambda	2.7e-5 / 98.17
22	Hypothetical protein	+	13148..13510	363	-	TPA: MAG TPA: hypothetical protein [Bacteriophage sp.]	0.002 / 31.20		
23	Hypothetical protein	+	13507..14073	567	Phage_Gp15 (pfam06854)	TPA: MAG TPA: hypothetical protein [*Siphoviridae* sp.]	3e-42 / 49.70		
24	Tape measure protein TMP	+	14126..16522	2397	Phage-related protein (COG5412)	TPA: MAG TPA: tail tape measure protein [*Siphoviridae* sp.]	5e-111 / 45.67	Tape measure protein TMP, *Staphylococcus aureus* phage 80alpha	4e-18 / 99.93
25	**Distal tail protein DIT**	+	16515.. 18968	2454	-	TPA: MAG TPA: distal tail protein [*Siphoviridae* sp.]	9e-27 / 35.35	Distal tail protein DIT, *Bacillus subtilis* phage SPP1	7.2e-17 / 99.76

26	**Fiber lower protein FibL**	+	18968.. 19426	459	-	hypothetical protein [Bacilli bacterium]	0.003 / 32.48	Fiber lower protein FibL, *Staphylococcus aureus* phage 80alpha	3.5 / 93.31
27	Tail-associated lysin TAL	+	19426..22239	2814	Prophage_tail (pfam06605)	hypothetical protein DBY08_04435 [*Clostridiales* bacterium]	1e-62 / 67.31	Tail-associated lysin TAL, *Staphylococcus aureus* phage 80alpha	2.1e-23 / 99.94
28	**Tail needle protein TNP**	+	22521..22841	321	-	hypothetical protein C1860_07715 [*Eggerthella lenta*]	4e-40 / 63.81	**Tail needle protein** TNP, *Escherichia coli* phage HK620	6.1e-5 / 98.16
29	Hypothetical protein	+	22855..23067	213	-	hypothetical protein CE91St30_02930 [*Raoultibacter timonensis*]	1e-11 / 62.69		
30	Amidase endolysin -<br>Dextran sucrase	+	23068..24045	978	Amidase_2 (pfam01510)	N-acetylmuramoyl-L-alanine amidase [Paraeggerthella hongkongensis]	0.0 / 63.27	Amidase endolysin<br>N-term: *Streptococcus pneumoniae*<br>Dextran sucrase<br>C-term: *Limosilactobacillus fermentum*	7.3e-14 / 99.7<br>6.6e-3 / 97.8
31	Hypothetical protein	+	24199..25059	861	-	hypothetical protein [*Atopobiaceae* bacterium]	4e-59 / 60.89		
32	Hypothetical protein	+	25179..25421	243	-	-			
33	Hypothetical protein	+	25519..26385	867	DUF1351 (pfam07083)	hypothetical protein [*Adlercreutzia caecimuris*]	3e-16 / 30.66		
34	DNA gyrase inhibitor YacG	+	26390..26578	189	-	hypothetical protein [*Lewinella* sp.]	0.002 / 60.71	DNA gyrase inhibitor YacG, *Escherichia coli*	1.1e-4 / 97.65
35	Single-stranded DNA-binding protein SSBP	+	26575..27027	453	RPA_2b-aaRSs_OBF_like (cl09930)	single-stranded DNA-binding protein [*Raoultibacter timonensis*]	1e-69 / 76.16	Single-stranded DNA-binding protein, *Escherichia coli*	3e-25 / 99.95
36	Hypothetical protein	+	27038..27331	294	-	-			
37	Hypothetical protein	+	27443..27727	285	-	TPA: MAG TPA: hypothetical protein [*Siphoviridae* sp.]	1e-30 / 59.57		
38	Hypothetical protein	+	27730..28056	327	-	-			
39	Replication initiator	+	28192..28950	759	-	hypothetical protein L6V86_08445	7e-25 / 35.33	Replication initiator protein DnaD,	6.2e-5 / 98.1

	protein DnaD					[*Treponema* sp.]		*Staphylococcus aureus*	
40	Helicase loader	+	28965..29783	819	P-loop_NTPase (cl38936)	ATP-binding protein [*Lachnospiraceae* bacterium]	5e-42 / 33.96	primase, helicase loader	2.4e-21 / 99.9
41	Repressor transcription regulator	+	29814..29948	135	-	-		Repressor transcription regulator, *Helicobacter pylori*	1.6e-10 / 99.28
42	Hypothetical protein	+	29961..30170	210	-	-			
43	Holliday junction resolvase RuvC	+	30167..30628	462	RuvC_like (cl21482)	hypothetical protein BN3658_00054 [*Coriobacteriaceae* bacterium CHKCI002]	8e-17 / 32.28	Holliday junction endodeoxyribonuclease RuvC, *Thermus thermophilus*	2.4e-23 / 99.93
44	MazG-like pyrophosphohydrolase	+	30628..30915	288	NTP-PPase (cl16941)	TPA: MAG TPA: nucleoside triphosphate pyrophosphohydrolase [*Siphoviridae* sp.]	0.014 / 44.23	MazG-like pyrophosphohydrolase, *Archeaoglobus fulgidus*	3.1e-11 / 99.32
